# Longitudinal spinal cord MRI in adult 5q-SMA: biomarker and pathophysiological insights

**DOI:** 10.1007/s00415-026-14013-7

**Published:** 2026-07-23

**Authors:** Felipe Franco da Graça, Thiago Junqueira Ribeiro de Rezende, Cristina Iwabe, Carelis González-Salazar, Rodrigo de Holanda Mendonça, Edmar Zanoteli, Marcondes Cavalcante França-Jr

**Affiliations:** 1 Department of Neurology, Faculdade de Ciências Médicas, Universidade Estadual de Campinas (UNICAMP), Sao Paulo, Brazil; 2 Department of Neurology, Faculdade de Medicina, Universidade de São Paulo (FMUSP), São Paulo, Brazil

**Keywords:** Spinal muscular atrophy, MRI, Spinal cord, Diffusion tensor imaging, Gray matter, Biomarker, Adult SMA

## Abstract

**Background:**

Spinal muscular atrophy (SMA) is a progressive neuromuscular disease caused by biallelic *SMN1* mutations and widespread lower motor neuron degeneration. Objective biomarkers to monitor progression and treatment response are critically needed. Spinal cord (SC) MRI, including diffusion tensor imaging (DTI), is a promising non-invasive approach, but data in adults remain scarce.

**Methods:**

We conducted an exploratory longitudinal study in 26 adults with 5q-SMA (type 2: *n* = 4; type 3: *n* = 22; median age 29.5 years) and 27 controls undergoing 3 T cervical SC MRI. Gray matter (GM) and total cross-sectional area (CSA) and DTI metrics (FA, MD, AD, RD) were extracted from three white matter tracts. Patients underwent motor (MFM-32, HFMSE, RULM), neurophysiological (CMAP, MUNIX) and fatigue assessments. 22 patients completed 12–14-month follow-up.

**Results:**

Total SC CSA was reduced across C2–C6 (Cohen d up to 1.02) but showed limited functional correlations. GM CSA was reduced in SMA versus controls (11.90 ± 2.56 vs. 13.92 ± 1.72 mm^2^; *p* = 0.001; Cohen *d* = 0.93) and correlated robustly with MFM-32 (*R* = 0.60), HFMSE (*R* = 0.56), RULM (*R* = 0.54) and CMAP (*R* = 0.46). DTI revealed FA reduction and markedly elevated MD in fasciculi gracilis and cuneatus (Cohen d up to 2.44), without significant RD changes, suggesting axonal pathology. No DTI metric correlated with clinical variables. No longitudinal changes were detected at 12–14 months.

**Conclusions:**

Selective GM CSA yields robust functional correlations, supporting its role as a candidate imaging biomarker and outperforming total CSA. DTI abnormalities in sensory tracts extend the known pathological scope of adult SMA beyond motor neurons. Longer follow-up is warranted to detect disease progression.

**Supplementary Information:**

The online version contains supplementary material available at 10.1007/s00415-026-14013-7.

## Introduction

Spinal muscular atrophy (SMA) is an autosomal recessive neuromuscular disease caused by biallelic pathogenic *SMN1* variants, leading to deficiency of the SMN protein and progressive degeneration of alpha motor neurons in the ventral horns of the spinal cord (SC) [1, 2]. The disease encompasses a clinical spectrum ranging from severe infantile forms (type 1) to milder adult-onset presentations (types 3 and 4), with intermediate forms (type 2) characterized by inability to walk independently. The therapeutic landscape has been transformed by the approval of three disease-modifying treatments—nusinersen, onasemnogene abeparvovec and risdiplam—which have substantially improved motor outcomes, particularly in younger and pre-symptomatic patients [1, 2].

Despite these advances, the assessment of disease course and treatment response in adult SMA patients remains challenging. Established clinical outcome measures such as the Hammersmith Functional Motor Scale Expanded (HFMSE), the Revised Upper Limb Module (RULM) and the Motor Function Measure (MFM32) reflect functional status but may lack sensitivity to detect slow or subclinical disease progression in adults with longstanding disease [3, 4]. There is therefore an increasing need for objective, quantitative biomarkers capable of capturing underlying neuropathological changes.

Advanced SC neuroimaging has emerged as a promising tool in this context. Gray matter (GM) cross-sectional area (CSA) measurement provides an in vivo index of anterior horn damage [5, 6], while diffusivity analyses enable the characterization of white matter (WM) microstructure through parameters such as fractional anisotropy (FA), axial diffusivity (AD), mean diffusivity (MD), and radial diffusivity (RD) [7–9]. Previous studies in adult SMA cohorts have reported spinal cord gray matter atrophy; however, white matter involvement in specific ascending and descending spinal cord tracts remains less extensively characterized. There is indeed preclinical evidence that SC structural changes may extend beyond motor pathways and include sensory tracts [10–12]. Furthermore, the precise clinical/neurophysiological correlates and the natural history of adult SMA-related SC damage still need investigation.

The present exploratory study was designed to tackle these open questions relative to SC damage in SMA. The specific goals were (1) to characterize SC GM and WM abnormalities in adults with 5q-SMA compared to healthy controls; (2) to assess correlations between SC imaging parameters and validated motor, neurophysiological and fatigue outcome measures; and (3) to evaluate longitudinal course of these imaging measures over 12–14 months.

## Methods

### Participants

Adults with genetically confirmed 5q-SMA (types 2 and 3) were prospectively recruited at the University of Campinas (UNICAMP). The inclusion criteria were age ≥ 18 years; confirmed biallelic *SMN1* variants; clinical diagnosis of SMA type 2 or type 3.

Exclusion criteria included contraindications to MRI and inability to provide written informed consent. Healthy controls (HCs) were recruited concurrently. Controls had no personal or family history of neurological disease. All of them had normal neurological examination. The study was approved by the Ethics Committee of Universidade Estadual de Campinas (CAAE 29538820.0.0000.5404) and was conducted in accordance with the principles of the Declaration of Helsinki. All participants provided written informed consent.

Participants are assessed at 2 time points 1 year apart. On each visit, clinical, neurophysiological, and imaging data were acquired, as detailed below. Data collection took place between 2021 and 2023.

### Clinical and neurophysiological assessments

At each time point, patients underwent structured clinical assessment including motor function scales (Hammersmith Functional Motor Scale Expanded [HFMSE], Revised Upper Limb Module [RULM], and the 32-item Motor Function Measure [MFM-32] with subscores Standing Position and Transfers [D1], Axial and Proximal Motor Function [D2], and Distal Motor Function [D3]) [13–15] and fatigue scales (Fatigue Severity Scale [FSS] and Modified Fatigue Impact Scale [MFIS] with physical, cognitive, and psychosocial subscores) [16, 17]. All scales were performed by the same trained physical therapist on the same day MRIs were acquired; the physical therapist was blinded to all imaging results. Neurophysiological evaluations were performed by a board-certified clinical neurophysiologist using commercially available electromyography equipment (Neurosoft, Neuro-MEP-Micro, Ivanovo, Russia) according to established guidelines [18]. The retrieved parameters were the amplitudes of the compound muscle action potential (CMAP) of the right abductor digiti minimi and the Motor Unit Number Index (MUNIX) of the same muscle. For each visit, three technically acceptable MUNIX measurements were obtained, and the median value was used for analysis.

### MRI acquisition

All participants underwent MRI on a 3 T Philips Achieva 3 T MRI scanner equipped with a 16-channel neurovascular coil for spinal cord imaging. Standard T1- and T2-weighted sequences were also acquired to screen for incidental abnormalities unrelated to the disease (e.g., compressive myelopathy). The imaging protocol included the following sequences:

- 3D T2-weighted imaging: repetition time (TR)/echo time (TE) = 3500/120 ms; flip angle = 90°; number of averages = 1; voxel size = 0.8 × 0.8 × 0.8 mm^3^; field of view (FOV) = 256 × 256 × 192 mm^3^; bandwidth = 389.4 Hz/pixel; phase encoding direction = anterior-to-posterior (A >  > P). Vertebral levels: C2–C5.
- Fat-suppressed 3D slab-selective fast field echo (3D-FFE): TR/TE = 23/5 ms; flip angle = 7°; FOV = 240 × 180 × 15 mm^3^; voxel size = 0.5 × 0.5 × 5 mm^3^; number of excitations (NEX) = 8; 10 contiguous axial slices; phase encoding direction = A >  > P.
- Diffusion-weighted imaging (DWI): TR/TE = 3429/70 ms; flip angle = 90°; number of averages = 1; FOV = 65 × 64 × 15 mm^3^; voxel size = 0.88 × 0.88 × 5.0 mm^3^; 32 diffusion-encoding directions; maximum b-value = 800 s/mm^2^; phase encoding direction = A >  > P.

### MRI analysis

Spinal cord imaging analyses were performed using the Spinal Cord Toolbox v.6.2 [19].

#### Spinal cord T2w

**Fig. 1:**
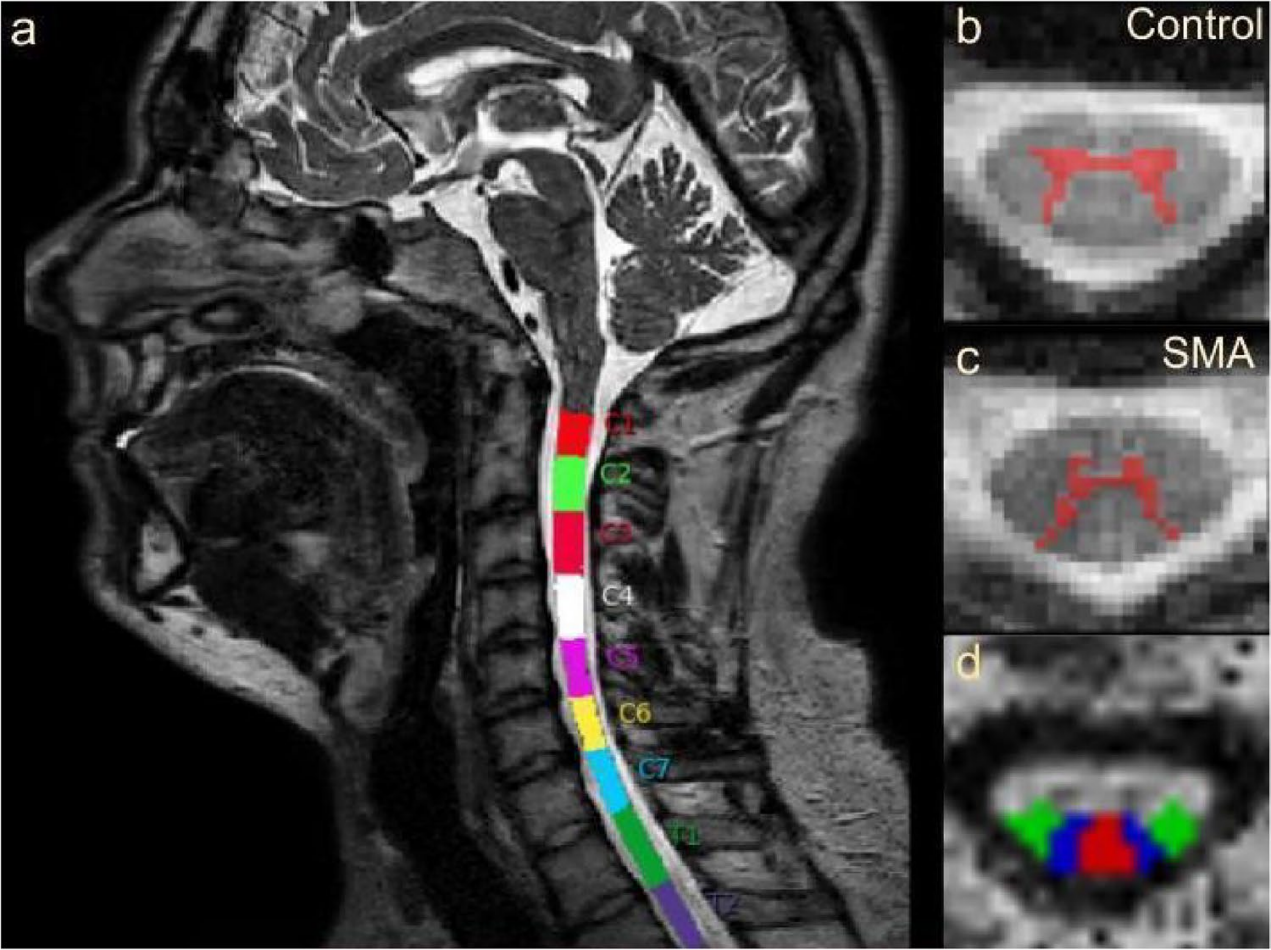
**a** Sagittal T2-weighted image illustrating cervical spinal cord segmentation across vertebral levels; **b** axial T2*-weighted image of the spinal cord in a healthy control demonstrating gray matter cross-sectional area (CSA); **c** axial T2*-weighted image of the spinal cord in a patient, showing visually reduced gray matter CSA compared with the control. **d** Axial diffusion tensor imaging fractional anisotropy (DTI-FA) map depicting the white matter tracts analyzed: gracile fasciculus (red), cuneate fasciculus (blue), and lateral corticospinal tract (green)

Total spinal cord cross-sectional area (CSA) was quantified using an automated deep learning-based segmentation approach available in the Spinal Cord Toolbox (SCT) [20]. All segmentations underwent visual quality control and were manually refined when required prior to vertebral level labeling [21] (Fig. 1). Subsequently, each subject’s image was registered to the PAM50 template through a combination of affine and non-linear transformations [22]. The template was then propagated into individual subject space to enable extraction of mean CSA values across predefined vertebral levels. To account for spinal cord curvature, CSA measurements were adjusted based on the angle between each axial slice and the corresponding spinal cord centerline [19].

#### Spinal cord T2*w

Spinal cord and gray matter (GM) were automatically segmented using deep learning-based methods [23], followed by visual inspection and manual correction when required. All T2*-weighted images were subsequently registered to the PAM50 template using established procedures [22, 24, 25], after which the template was transformed into individual subject space (Fig. 1). Mean GM cross-sectional area (CSA) was then calculated and adjusted for spinal cord curvature based on the angle between each slice and the spinal cord centerline. In contrast to the T2-weighted analysis, GM CSA values were averaged across all available vertebral levels (C2 to C5), as the T2*-weighted acquisition is inherently noisier and limited to 15 slices.

#### Spinal cord DWI

Diffusion-weighted spinal cord data were preprocessed using a standardized pipeline. Denoising was performed with the Marchenko–Pastur principal component analysis (MP-PCA) method [26], followed by suppression of Gibbs ringing artifacts through subvoxel-shift correction as implemented in MRtrix3 [27, 28]. Motion correction was applied using the Spinal Cord Toolbox (SCT, version 6.2) [19]. To address susceptibility-related geometric distortions, the motion-corrected diffusion images were co-registered to each subject’s native T2-weighted spinal cord image using SCT’s robust registration framework. Diffusion tensor metrics, including fractional anisotropy (FA), mean diffusivity (MD), axial diffusivity (AD), and radial diffusivity (RD), were derived using a robust tensor fitting approach with outlier rejection [29].

For level-specific quantification at the cervical cord (C2–C5), the PAM50 template [22] was nonlinearly aligned to each subject’s diffusion space. This registration was initialized using deformation fields obtained from the prior PAM50-to-T2 alignment performed during anatomical processing, ensuring accurate vertebral level correspondence and segmentation of spinal cord regions. Diffusion metrics were extracted bilaterally from major white matter tracts, including the lateral corticospinal tract, fasciculus gracilis, and fasciculus cuneatus.

### Statistical analysis

Prior to statistical analyses, the distribution of all variables was evaluated using the Kolmogorov–Smirnov test. As most measures approximated a normal distribution, parametric statistical approaches were applied. SMA patients were compared with age- and sex-matched control participants for both MRI-derived metrics and demographic variables. Cross-sectional group differences were assessed using analysis of covariance (ANCOVA). Potential confounding effects of age and sex were accounted for by adjusting all variables accordingly prior to analysis. For longitudinal analyses, a one-way repeated-measures ANOVA was used to evaluate changes between baseline and follow-up within the patient group. Pearson correlation coefficients were calculated to assess associations between imaging metrics and clinical and neurophysiological variables. Multiple comparisons across spinal levels and structures were corrected using Bonferroni correction. Effect sizes were reported as Cohen’s d. Statistical significance was set at p < 0.05 after Bonferroni correction. Statistical analyses were performed using MATLAB R2022b (MathWorks, Natick, MA, USA).

## Results

### Participants

Twenty-six patients (type 2: *n* = 4; type 3: *n* = 22) and 27 HCs were enrolled at baseline. Longitudinal follow-up (12–14 months after baseline) was completed by 22 patients.

Demographic and clinical characteristics for patients and controls at baseline are presented in Table 1.

**Table 1:** Baseline demographic and clinical characteristics of patients and healthy controls

Characteristic	Patients (*n* = 26)	Controls (*n* = 27)	*p*-value
Age, years, median (IQR)	29.5 (23.9–37.1)	33.6 (30.3–41.6)	0.2
Sex (M/F)	17/9	15/12	0.6
Disease duration, years, median (IQR)	26.0 (20.0–30.1)	—	—
SMA type (type 2/type 3)	4/22	—	—
Ambulation (ambulatory/non-ambulatory)	6/20	—	—
On nusinersen at baseline/follow-up	3/6	—	—
SMN2 copies (2 copies/3 copies/4 copies)	3*/18/5		
Patients completing T2 follow-up	22	—	—

^*^—Compound heterozygous patients

### Total spinal cord cross-sectional area

Total spinal cord CSA was significantly reduced in SMA patients compared to healthy controls at multiple cervical levels, as detailed in Table 2. Correlations between total CSA and clinical or neurophysiological outcomes were not significant at any cervical level, with the exception of association with MFM D3 at C3 level (*R* = 0.53; *p*-Bonferroni = 0.044).

**Table 2:** Comparison of total spinal cord CSA between SMA patients and healthy controls by cervical level. Bold *p*-values indicate significance after Bonferroni correction (*p* < 0.05)

Level	Patients (mean ± SD, mm^2^)	Controls (mean ± SD, mm^2^)	*p*-value (Bonferroni)	Cohen’s d
Total CSA				
C1	69.99 ± 6.12	74.11 ± 7.57	0.056	0.60
C2	67.10 ± 11.47	73.42 ± 8.89	**0.020**	0.62
C3	66.17 ± 12.46	76.13 ± 9.21	**0.001**	0.91
C4	68.24 ± 13.10	79.62 ± 8.90	** < 0.001**	1.02
C5	64.30 ± 12.57	74.75 ± 9.80	**0.004**	0.93
C6	55.35 ± 12.39	65.27 ± 10.16	**0.031**	0.88
C7	45.15 ± 13.62	53.13 ± 8.55	0.145	0.70
WM-CSA	56.91 ± 8.30	62.00 ± 7.97	0.064	0.63
GM CSA	11.90 ± 2.56	13.92 ± 1.72	**0.002**	0.93

### Gray matter cross-sectional area

**Fig. 2:**
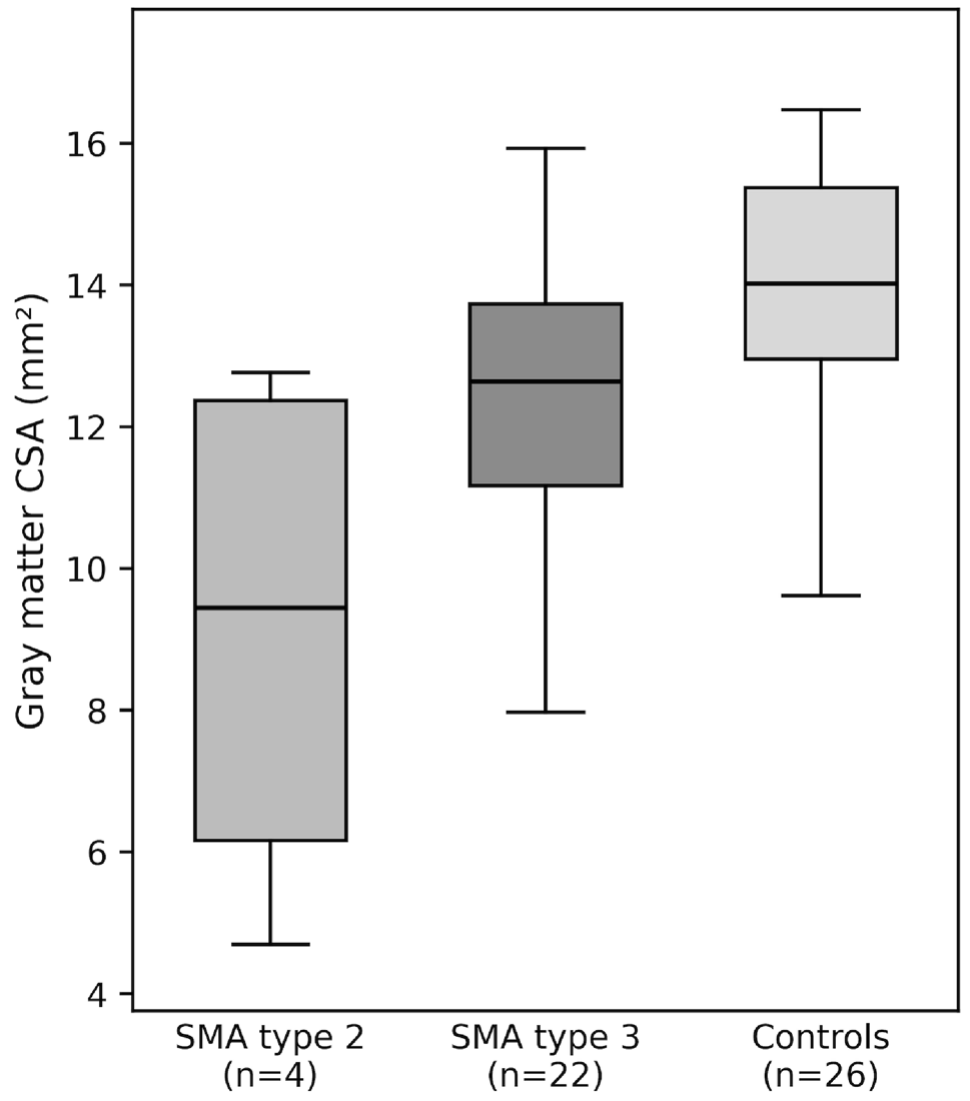
Gray matter cross-sectional area across groups. Box plots show gray matter CSA in SMA type 2, SMA type 3, and controls. Boxes represent the interquartile range, central lines the median, and whiskers the minimum and maximum values

Spinal cord GM CSA was significantly reduced in SMA patients compared to healthy controls (11.90 ± 2.56 mm^2^ vs. 13.92 ± 1.72 mm^2^; *p* = 0.002; Cohen *d* = 0.93), consistent with anterior horn motor neuron pathology (Table 2, Fig. 2).

GM CSA correlated significantly with multiple validated motor function scales (Table 3). The strongest associations were observed with MFM D2 (*R* = 0.61, *p* = 0.0016), MFM-32 total (*R* = 0.60, *p* = 0.0018) and HFMSE (*R* = 0.56, *p* = 0.0046). Significant correlations were also found with RULM (*R* = 0.54, *p* = 0.0065), MFM D3 (*R* = 0.55, *p* = 0.0058) and MFM D1 (*R* = 0.43, *p* = 0.040).

**Table 3:** Correlations between GM CSA and clinical and neurophysiological variables

Clinical variable	*R* (Pearson)	*p*-value
MFM-32 total	0.60	0.0018
MFM D1	0.43	0.040
MFM D2	0.61	0.0016
MFM D3	0.55	0.0058
HFMSE	0.56	0.0046
RULM	0.54	0.0065
CMAP (abductor digiti minimi)	0.46	0.019
MUNIX (median)	0.24	0.24
Disease duration	− 0.15	0.47
FSS (fatigue)	0.14	0.53
MFIS total (fatigue)	0.22	0.29

### Diffusion tensor imaging

DTI analysis revealed microstructural white matter abnormalities predominantly affecting sensory tracts but also in the lateral corticospinal tracts.

FA was significantly reduced in patients compared to controls after Bonferroni correction in four structures: fasciculus gracilis left side at C3 level (*p* = 0.010; Cohen *d* = 1.06), fasciculus gracilis right side at C3 level (*p* = 0.006; Cohen *d* = 1.15), fasciculus cuneatus left side at C3 level (*p* = 0.012; Cohen *d* = 1.03) and lateral corticospinal tract right side at level C3 (*p* = 0.014; Cohen *d* = 1.24).

MD was markedly elevated in patients across sensory tracts with the most robust effects in fasciculus gracilis right at C3 (*p*-Bonferroni < 0.001; Cohen *d* = 2.44), C4 (Cohen *d* = 2.31; *p*-Bonferroni < 0.001) and C5 (*p*-Bonferroni = 0.002; Cohen *d* = 1.65), fasciculus gracilis left at C3 level (*p*-Bonferroni = 0.035; Cohen *d* = 1.33) and fasciculus cuneatus right side at C3 level (*p*-Bonferroni = 0.015; Cohen *d* = 1.38). Lateral corticospinal tract right side at C4 level was elevated as well (*p*-Bonferroni = 0.045; Cohen *d* = 1.20).

AD showed elevation in fasciculus cuneatus right side at level C4 (*p*-Bonferroni = 0.039; Cohen *d* = 1.33) and lateral corticospinal tract left side at C5 level (*p*-Bonferroni = 0.017; Cohen *d* = 1.26). Importantly, RD showed no significant differences between patients and controls across all structures and levels (all *p* > 0.12), indicating an absence of predominant myelin pathology and suggesting that the observed changes reflect primarily axonal involvement (supplementary Table 1).

No significant correlations were observed between DTI metrics in any white matter tract and clinical or neurophysiological variables, including motor function scales (HFMSE, RULM, MFM-32), CMAP, MUNIX, fatigue scales or disease duration (all *p* > 0.05 after Bonferroni correction).

### Longitudinal analysis

No significant changes were observed in any MRI parameter between baseline and follow-up, including total CSA in all levels (*p* > 0.18 uncorrected; Cohen *d* < 0.38), GM CSA (baseline: 11.90 ± 2.56 mm^2^ vs. follow-up: 12.16 ± 2.71 mm^2^; *p* = 0.73; Cohen *d* = 0.10), FA, AD, MD or RD across all tracts and levels (supplementary Table 2). Similarly, no significant longitudinal changes were detected in clinical or neurophysiological parameters (HFMSE, RULM, MFM, CMAP, MUNIX). Subgroup analysis comparing nusinersen-treated patients to treatment-naive patients did not reveal significant differences in any imaging or clinical variable at either time point or in the magnitude of longitudinal change.

## Discussion

This longitudinal observational study investigated spinal cord morphometric and microstructural SC MRI changes in adults with 5q-SMA. The main findings were significant total spinal cord CSA reduction across cervical levels with limited functional correlations, significant GM CSA reduction with robust correlations with multiple motor function tests and CMAP, but not with fatigue scales; DTI abnormalities in sensory WM tracts and lateral corticospinal tract characterized by reduced FA and markedly elevated MD without significant RD changes; and absence of significant longitudinal changes in imaging or clinical parameters over 12–14 months.

### Total spinal cord CSA

Total spinal cord CSA was significantly reduced in SMA patients compared to controls at multiple cervical levels (C2–C6) after Bonferroni correction, with large effect sizes (Cohen d ranging from 0.62 to 1.02), indicating widespread structural atrophy of the spinal cord. The largest effect size was observed at C4, suggesting a mid-cervical predominance of total cord atrophy. This distribution is anatomically compatible with the involvement of cervical segments contributing to proximal upper-limb innervation, paralleling the typical proximal pattern of weakness observed in SMA [1, 2]. These findings are consistent with the diffuse nature of SMA-related neurodegeneration and extend prior reports of cervical spinal cord atrophy in adult SMA cohorts [6]. Because this level-wise pattern was assessed for total CSA only, it cannot be directly extrapolated to the gray matter compartment, which was analyzed as an aggregate measure. Moreover, despite this robust group-level difference, total CSA showed very limited associations with clinical and neurophysiological outcomes. The only significant correlation after correction was with MFM D3 at C3. This dissociation between structural atrophy and functional correlates underscores a key limitation of total CSA as a biomarker: it combines gray and white matter compartments, thereby diluting the changes derived from the anterior horn, which is the primary locus of pathology in SMA.

### Gray matter atrophy as a motor biomarker

The significant reduction in SC GM CSA in SMA patients, with a large effect size (Cohen *d* = 0.93), is consistent with the established neuropathology of SMA, in which loss of anterior horn alpha motor neurons constitutes the primary substrate of disability [1, 2]. Our findings align with previous reports of cervical spinal cord atrophy in adult SMA, but extend them by showing broader functional correlates of selective GM CSA. El Mendili et al. documented cervical cord atrophy using total CSA and radial distance measurements [6], but did not perform separate GM segmentation and found no significant correlations with clinical measures. Querin et al. later demonstrated cervical GM atrophy in adult type III/IV SMA [5], although significant clinical associations were limited to deltoid strength. In contrast, GM CSA in our cohort correlated with multiple motor outcomes, including MFM-32, HFMSE and RULM, as well as CMAP. This difference may partly reflect the broader functional spectrum of our cohort, which included type 2 and type 3 adults with a higher proportion of non-ambulatory patients, as well as the use of outcome measures particularly relevant to more severely affected individuals [4–6, 30]

The present study extends these observations by demonstrating robust and consistent correlations between GM CSA and a broader set of outcome measures, encompassing all three MFM domains, HFMSE, RULM, and CMAP, with correlation coefficients ranging from 0.43 to 0.61. These associations reinforce that GM CSA reflects the cumulative burden of motor neuron loss across functional domains and supports its role as a clinically meaningful imaging biomarker in adult SMA.

### White matter pathology in sensory tracts

A relevant finding of this study was the presence of DTI abnormalities in sensory white matter tracts, particularly the fasciculi gracilis and cuneatus. Reduced FA and markedly elevated MD suggest microstructural abnormalities, while the absence of significant RD changes is more compatible with predominant axonal involvement rather than demyelination.

Our results differ from those of Querin et al. [5], who reported preserved spinal white matter integrity in adult SMA. This discrepancy may be partly related to differences in regional assessment and cohort composition. Querin et al. evaluated broader template-based white matter regions, including the dorsal columns as a composite region, whereas our study separately assessed the fasciculus gracilis, fasciculus cuneatus across cervical levels. This tract-specific approach may have increased sensitivity to detect subtle sensory pathway abnormalities. In addition, our cohort included a higher proportion of non-ambulatory patients, representing a clinically more affected group. However, because DTI metrics did not correlate with functional measures, a direct relationship between white matter abnormalities and clinical severity cannot be inferred.

These findings suggest that structural spinal cord involvement in adult SMA may extend beyond the classical lower motor neuron compartment. Preclinical evidence supports the notion that SMN deficiency may affect sensory neurons: nociceptive dorsal root ganglion neurons have been shown to be hyperexcitable in SMA mouse models through a signaling cascade involving elevated norepinephrine, NF-κB activation and upregulation of voltage-gated sodium channels [11]. Furthermore, in a zebrafish model of SMA, DRG neurons developed abnormally as a secondary consequence of motoneuron dysfunction through a non-cell-autonomous mechanism [12]. The absence of significant correlations between DTI metrics and clinical or neurophysiological variables suggests that sensory tract involvement in adult SMA may represent a subclinical structural phenomenon, not captured by currently available outcome measures, which are predominantly designed to assess motor function. Whether these imaging abnormalities reflect primary sensory neuron pathology driven by SMN deficiency, or a secondary structural consequence of motor neuron degeneration, remains to be determined. Future studies incorporating dedicated sensory assessments, such as quantitative sensory testing or sensory nerve conduction studies, may help clarify the functional relevance of these white matter changes.

In this context, the recent report of peripheral sensory neuropathy as an adverse event in participants in the STEER trial of intrathecal onasemnogene abeparvovec [31] raises a clinically relevant question: whether pre-existing structural sensory tract abnormalities in adult SMA could reflect a biological substrate of increased neuronal vulnerability, potentially influencing susceptibility to treatment-related sensory events. We emphasize that our data do not establish a causal link, and this hypothesis requires dedicated investigation in future studies.

### Longitudinal stability

No significant longitudinal changes were detected in any imaging or clinical parameter over 12–14 months, including in the subgroup analyses by treatment status. This is consistent with the known slow progression of adult SMA types 2 and 3 and with prior longitudinal imaging studies. Savini et al. reported stable SC CSA over 21 months of nusinersen treatment in adult SMA 3a patients [9], while Gallone et al. found no significant change in muscle fat fraction over 14 months despite clinical improvement [32]. Collectively, these findings suggest that a 12–14-month follow-up period is insufficient to detect imaging changes in adult SMA and that future studies should extend to at least 24–36 months.

### Limitations

Several limitations should be acknowledged. First, this is an exploratory study with a relatively small sample size (*n* = 26), limiting statistical power and generalizability, particularly for subgroup analyses. Second, the 12–14-month follow-up period may be insufficient to capture meaningful longitudinal change in this slowly progressive population. Third, the variable treatment status introduces heterogeneity that could not be fully controlled given the sample size. All findings should need replication in larger, prospective cohorts.

## Conclusions

This exploratory study provides evidence that adults with 5q-SMA exhibit significant total spinal cord CSA reduction across cervical levels, reflecting widespread structural atrophy. However, total CSA shows very limited functional correlations, with only a marginal association with MFM D3, highlighting its limitations as a biomarker. In contrast, selective GM CSA measurement yields robust and consistent correlations with multiple motor outcome measures, supporting its role as a clinically meaningful candidate imaging biomarker. This dissociation reinforces that the anterior horn, the primary locus of SMA pathology, must be specifically targeted to capture functionally relevant spinal cord changes. DTI abnormalities in sensory white matter tracts further indicate that structural spinal cord involvement extends beyond the classical lower motor neuron compartment, with potential implications for disease biology and therapeutic safety monitoring. No longitudinal changes were detected in any imaging parameter over 12–14 months, underscoring the need for longer follow-up in future studies.

## Supplementary Information

Below is the link to the electronic supplementary material.

Supplementary file1 (DOCX 52 KB)

## Data Availability

The datasets generated and/or analyzed during the current study are not publicly available due to patient privacy and institutional restrictions but are available from the corresponding author on reasonable request.
