# Supplementary material for: Longitudinal spinal cord MRI in adult 5q-SMA: biomarker and pathophysiological insights
Source: J Neurol. 2026 Jul 23;273(8):487. doi: 10.1007/s00415-026-14013-7 (PMC13395906; doi:10.1007/s00415-026-14013-7)
Supplement: Supplementary file 1 — Supplementary file1 (DOCX 52 KB) [file 415_2026_14013_MOESM1_ESM.docx]

Supplementary Table 1. Group comparisons of DTI metrics (FA, MD, AD, RD) between SMA patients and healthy controls across cervical spinal cord levels (C2–C5) and white matter tracts.

| Tract | Side | Level | Controls (mean ± SD) | SMA (mean ± SD) | p (uncorrected) | p (Bonferroni) | Cohen's d |
| --- | --- | --- | --- | --- | --- | --- | --- |
| FA | | | | | | | |
| Fasciculus Gracilis | Left | C2 | 0.81320 ± 0.06452 | 0.77079 ± 0.07008 | 0.083 | 1.000 | 0.63 |
| Fasciculus Gracilis | Left | C3 | 0.83596 ± 0.04663 | 0.77717 ± 0.06368 | <0.001 | 0.011 | 1.06 |
| Fasciculus Gracilis | Left | C4 | 0.81048 ± 0.05893 | 0.79358 ± 0.04866 | 0.107 | 1.000 | 0.31 |
| Fasciculus Gracilis | Left | C5 | 0.79612 ± 0.06486 | 0.78317 ± 0.07098 | 0.350 | 1.000 | 0.19 |
| Fasciculus Gracilis | Right | C2 | 0.82260 ± 0.06225 | 0.76327 ± 0.09822 | 0.031 | 0.748 | 0.72 |
| Fasciculus Gracilis | Right | C3 | 0.83513 ± 0.04940 | 0.76365 ± 0.07318 | <0.001 | 0.006 | 1.15 |
| Fasciculus Gracilis | Right | C4 | 0.81124 ± 0.04455 | 0.78086 ± 0.06272 | 0.020 | 0.484 | 0.56 |
| Fasciculus Gracilis | Right | C5 | 0.80487 ± 0.06239 | 0.78044 ± 0.07924 | 0.167 | 1.000 | 0.35 |
| Fasciculus Cuneatus | Left | C2 | 0.87948 ± 0.05125 | 0.83508 ± 0.10187 | 0.030 | 0.715 | 0.55 |
| Fasciculus Cuneatus | Left | C3 | 0.90035 ± 0.04370 | 0.83906 ± 0.07274 | <0.001 | 0.013 | 1.03 |
| Fasciculus Cuneatus | Left | C4 | 0.84735 ± 0.04901 | 0.82367 ± 0.05671 | 0.098 | 1.000 | 0.45 |
| Fasciculus Cuneatus | Left | C5 | 0.80908 ± 0.07612 | 0.78871 ± 0.08389 | 0.426 | 1.000 | 0.26 |
| Fasciculus Cuneatus | Right | C2 | 0.86193 ± 0.06398 | 0.82924 ± 0.08244 | 0.119 | 1.000 | 0.44 |
| Fasciculus Cuneatus | Right | C3 | 0.88625 ± 0.04244 | 0.83197 ± 0.08129 | 0.003 | 0.061 | 0.84 |
| Fasciculus Cuneatus | Right | C4 | 0.84073 ± 0.06038 | 0.83417 ± 0.06635 | 0.438 | 1.000 | 0.10 |
| Fasciculus Cuneatus | Right | C5 | 0.79476 ± 0.07767 | 0.78399 ± 0.06999 | 0.941 | 1.000 | 0.14 |
| Lateral Corticospinal Tract | Left | C2 | 0.80577 ± 0.05682 | 0.75454 ± 0.06421 | 0.042 | 1.000 | 0.85 |
| Lateral Corticospinal Tract | Left | C3 | 0.82298 ± 0.04370 | 0.76498 ± 0.06231 | 0.008 | 0.182 | 1.08 |
| Lateral Corticospinal Tract | Left | C4 | 0.77099 ± 0.05099 | 0.74086 ± 0.06136 | 0.084 | 1.000 | 0.54 |
| Lateral Corticospinal Tract | Left | C5 | 0.74467 ± 0.06262 | 0.73600 ± 0.07100 | 0.980 | 1.000 | 0.13 |
| Lateral Corticospinal Tract | Right | C2 | 0.80773 ± 0.05472 | 0.75256 ± 0.07783 | 0.020 | 0.468 | 0.82 |
| Lateral Corticospinal Tract | Right | C3 | 0.83090 ± 0.05450 | 0.74638 ± 0.07929 | <0.001 | 0.014 | 1.25 |
| Lateral Corticospinal Tract | Right | C4 | 0.78347 ± 0.05065 | 0.74190 ± 0.04368 | 0.016 | 0.395 | 0.88 |
| Lateral Corticospinal Tract | Right | C5 | 0.75728 ± 0.05742 | 0.71461 ± 0.06855 | 0.074 | 1.000 | 0.68 |
| MD | | | | | | | |
| Fasciculus Gracilis | Left | C2 | 0.00079 ± 0.00011 | 0.00088 ± 0.00017 | 0.388 | 1.000 | 0.65 |
| Fasciculus Gracilis | Left | C3 | 0.00073 ± 0.00011 | 0.00086 ± 0.00008 | 0.001 | 0.035 | 1.33 |
| Fasciculus Gracilis | Left | C4 | 0.00075 ± 0.00008 | 0.00082 ± 0.00008 | 0.028 | 0.663 | 0.87 |
| Fasciculus Gracilis | Left | C5 | 0.00074 ± 0.00009 | 0.00083 ± 0.00011 | 0.017 | 0.405 | 0.93 |
| Fasciculus Gracilis | Right | C2 | 0.00072 ± 0.00011 | 0.00088 ± 0.00016 | 0.010 | 0.241 | 1.18 |
| Fasciculus Gracilis | Right | C3 | 0.00065 ± 0.00009 | 0.00087 ± 0.00009 | <0.001 | <0.001 | 2.44 |
| Fasciculus Gracilis | Right | C4 | 0.00067 ± 0.00007 | 0.00083 ± 0.00007 | <0.001 | <0.001 | 2.31 |
| Fasciculus Gracilis | Right | C5 | 0.00065 ± 0.00010 | 0.00084 ± 0.00012 | <0.001 | 0.002 | 1.65 |
| Fasciculus Cuneatus | Left | C2 | 0.00072 ± 0.00011 | 0.00080 ± 0.00015 | 0.370 | 1.000 | 0.63 |
| Fasciculus Cuneatus | Left | C3 | 0.00065 ± 0.00009 | 0.00075 ± 0.00009 | 0.013 | 0.323 | 1.12 |
| Fasciculus Cuneatus | Left | C4 | 0.00067 ± 0.00007 | 0.00074 ± 0.00007 | 0.005 | 0.126 | 1.05 |
| Fasciculus Cuneatus | Left | C5 | 0.00065 ± 0.00010 | 0.00074 ± 0.00015 | 0.116 | 1.000 | 0.73 |
| Fasciculus Cuneatus | Right | C2 | 0.00073 ± 0.00012 | 0.00083 ± 0.00016 | 0.296 | 1.000 | 0.66 |
| Fasciculus Cuneatus | Right | C3 | 0.00065 ± 0.00008 | 0.00079 ± 0.00012 | <0.001 | 0.015 | 1.38 |
| Fasciculus Cuneatus | Right | C4 | 0.00068 ± 0.00007 | 0.00075 ± 0.00007 | 0.018 | 0.428 | 1.01 |
| Fasciculus Cuneatus | Right | C5 | 0.00067 ± 0.00009 | 0.00076 ± 0.00012 | 0.091 | 1.000 | 0.82 |
| Lateral Corticospinal Tract | Left | C2 | 0.00076 ± 0.00010 | 0.00085 ± 0.00014 | 0.212 | 1.000 | 0.75 |
| Lateral Corticospinal Tract | Left | C3 | 0.00073 ± 0.00009 | 0.00082 ± 0.00012 | 0.050 | 1.000 | 0.94 |
| Lateral Corticospinal Tract | Left | C4 | 0.00076 ± 0.00008 | 0.00083 ± 0.00007 | 0.007 | 0.167 | 1.07 |
| Lateral Corticospinal Tract | Left | C5 | 0.00074 ± 0.00010 | 0.00084 ± 0.00010 | 0.012 | 0.294 | 0.99 |
| Lateral Corticospinal Tract | Right | C2 | 0.00078 ± 0.00013 | 0.00087 ± 0.00017 | 0.504 | 1.000 | 0.58 |
| Lateral Corticospinal Tract | Right | C3 | 0.00073 ± 0.00010 | 0.00084 ± 0.00013 | 0.024 | 0.577 | 1.04 |
| Lateral Corticospinal Tract | Right | C4 | 0.00075 ± 0.00007 | 0.00084 ± 0.00007 | 0.002 | 0.045 | 1.21 |
| Lateral Corticospinal Tract | Right | C5 | 0.00075 ± 0.00010 | 0.00085 ± 0.00010 | 0.034 | 0.808 | 0.95 |
| AD | | | | | | | |
| Fasciculus Gracilis | Left | C2 | 0.00185 ± 0.00013 | 0.00196 ± 0.00027 | 0.730 | 1.000 | 0.52 |
| Fasciculus Gracilis | Left | C3 | 0.00178 ± 0.00013 | 0.00191 ± 0.00015 | 0.043 | 1.000 | 0.96 |
| Fasciculus Gracilis | Left | C4 | 0.00175 ± 0.00010 | 0.00186 ± 0.00016 | 0.046 | 1.000 | 0.89 |
| Fasciculus Gracilis | Left | C5 | 0.00167 ± 0.00012 | 0.00183 ± 0.00015 | 0.004 | 0.095 | 1.20 |
| Fasciculus Gracilis | Right | C2 | 0.00188 ± 0.00018 | 0.00195 ± 0.00022 | 0.668 | 1.000 | 0.35 |
| Fasciculus Gracilis | Right | C3 | 0.00180 ± 0.00013 | 0.00189 ± 0.00016 | 0.352 | 1.000 | 0.65 |
| Fasciculus Gracilis | Right | C4 | 0.00175 ± 0.00010 | 0.00186 ± 0.00015 | 0.058 | 1.000 | 0.88 |
| Fasciculus Gracilis | Right | C5 | 0.00170 ± 0.00013 | 0.00185 ± 0.00019 | 0.046 | 1.000 | 0.94 |
| Fasciculus Cuneatus | Left | C2 | 0.00185 ± 0.00015 | 0.00197 ± 0.00022 | 0.793 | 1.000 | 0.63 |
| Fasciculus Cuneatus | Left | C3 | 0.00174 ± 0.00014 | 0.00184 ± 0.00022 | 0.546 | 1.000 | 0.51 |
| Fasciculus Cuneatus | Left | C4 | 0.00165 ± 0.00010 | 0.00177 ± 0.00015 | 0.007 | 0.163 | 0.96 |
| Fasciculus Cuneatus | Left | C5 | 0.00152 ± 0.00016 | 0.00167 ± 0.00022 | 0.075 | 1.000 | 0.83 |
| Fasciculus Cuneatus | Right | C2 | 0.00183 ± 0.00015 | 0.00199 ± 0.00023 | 0.255 | 1.000 | 0.78 |
| Fasciculus Cuneatus | Right | C3 | 0.00172 ± 0.00012 | 0.00189 ± 0.00021 | 0.016 | 0.382 | 1.01 |
| Fasciculus Cuneatus | Right | C4 | 0.00165 ± 0.00012 | 0.00181 ± 0.00013 | 0.002 | 0.038 | 1.33 |
| Fasciculus Cuneatus | Right | C5 | 0.00152 ± 0.00014 | 0.00169 ± 0.00019 | 0.006 | 0.138 | 1.10 |
| Lateral Corticospinal Tract | Left | C2 | 0.00174 ± 0.00013 | 0.00181 ± 0.00020 | 0.790 | 1.000 | 0.40 |
| Lateral Corticospinal Tract | Left | C3 | 0.00168 ± 0.00013 | 0.00176 ± 0.00021 | 0.435 | 1.000 | 0.48 |
| Lateral Corticospinal Tract | Left | C4 | 0.00161 ± 0.00011 | 0.00173 ± 0.00012 | 0.007 | 0.157 | 1.03 |
| Lateral Corticospinal Tract | Left | C5 | 0.00152 ± 0.00016 | 0.00170 ± 0.00012 | <0.001 | 0.018 | 1.26 |
| Lateral Corticospinal Tract | Right | C2 | 0.00177 ± 0.00016 | 0.00182 ± 0.00023 | 0.371 | 1.000 | 0.27 |
| Lateral Corticospinal Tract | Right | C3 | 0.00170 ± 0.00012 | 0.00176 ± 0.00024 | 0.938 | 1.000 | 0.31 |
| Lateral Corticospinal Tract | Right | C4 | 0.00164 ± 0.00011 | 0.00173 ± 0.00012 | 0.021 | 0.509 | 0.84 |
| Lateral Corticospinal Tract | Right | C5 | 0.00157 ± 0.00016 | 0.00169 ± 0.00010 | 0.064 | 1.000 | 0.88 |
| RD | | | | | | | |
| Fasciculus Gracilis | Left | C2 | 0.00032 ± 0.00035 | 0.00036 ± 0.00018 | 0.679603 | 1 | 0.115 |
| Fasciculus Gracilis | Left | C3 | 0.00028 ± 0.00039 | 0.00036 ± 0.00014 | 0.33069 | 1 | 0.272 |
| Fasciculus Gracilis | Left | C4 | 0.00031 ± 0.00033 | 0.00032 ± 0.00011 | 0.965491 | 1 | 0.012 |
| Fasciculus Gracilis | Left | C5 | 0.00033 ± 0.00036 | 0.00033 ± 0.00013 | 0.946604 | 1 | 0.018 |
| Fasciculus Gracilis | Right | C2 | 0.00032 ± 0.00037 | 0.00037 ± 0.00022 | 0.525962 | 1 | 0.178 |
| Fasciculus Gracilis | Right | C3 | 0.00028 ± 0.00039 | 0.00038 ± 0.00020 | 0.224392 | 1 | 0.335 |
| Fasciculus Gracilis | Right | C4 | 0.00031 ± 0.00030 | 0.00032 ± 0.00011 | 0.880543 | 1 | 0.041 |
| Fasciculus Gracilis | Right | C5 | 0.00033 ± 0.00037 | 0.00032 ± 0.00013 | 0.832583 | 1 | 0.057 |
| Fasciculus Cuneatus | Left | C2 | 0.00024 ± 0.00036 | 0.00026 ± 0.00029 | 0.819929 | 1 | 0.066 |
| Fasciculus Cuneatus | Left | C3 | 0.00019 ± 0.00040 | 0.00023 ± 0.00015 | 0.686209 | 1 | 0.119 |
| Fasciculus Cuneatus | Left | C4 | 0.00024 ± 0.00031 | 0.00026 ± 0.00015 | 0.768925 | 1 | 0.08 |
| Fasciculus Cuneatus | Left | C5 | 0.00028 ± 0.00037 | 0.00027 ± 0.00015 | 0.865433 | 1 | 0.046 |
| Fasciculus Cuneatus | Right | C2 | 0.00025 ± 0.00035 | 0.00028 ± 0.00025 | 0.730337 | 1 | 0.097 |
| Fasciculus Cuneatus | Right | C3 | 0.00020 ± 0.00039 | 0.00027 ± 0.00024 | 0.444091 | 1 | 0.219 |
| Fasciculus Cuneatus | Right | C4 | 0.00025 ± 0.00029 | 0.00024 ± 0.00016 | 0.941357 | 1 | 0.02 |
| Fasciculus Cuneatus | Right | C5 | 0.00031 ± 0.00035 | 0.00027 ± 0.00012 | 0.657167 | 1 | 0.121 |
| Lateral Corticospinal Tract | Left | C2 | 0.00033 ± 0.00031 | 0.00039 ± 0.00015 | 0.386445 | 1 | 0.237 |
| Lateral Corticospinal Tract | Left | C3 | 0.00030 ± 0.00032 | 0.00038 ± 0.00015 | 0.274049 | 1 | 0.3 |
| Lateral Corticospinal Tract | Left | C4 | 0.00038 ± 0.00028 | 0.00042 ± 0.00018 | 0.558848 | 1 | 0.161 |
| Lateral Corticospinal Tract | Left | C5 | 0.00040 ± 0.00034 | 0.00040 ± 0.00012 | 0.997294 | 1 | 0.001 |
| Lateral Corticospinal Tract | Right | C2 | 0.00034 ± 0.00032 | 0.00039 ± 0.00019 | 0.442897 | 1 | 0.211 |
| Lateral Corticospinal Tract | Right | C3 | 0.00029 ± 0.00034 | 0.00042 ± 0.00023 | 0.128828 | 1 | 0.423 |
| Lateral Corticospinal Tract | Right | C4 | 0.00037 ± 0.00030 | 0.00040 ± 0.00009 | 0.654736 | 1 | 0.121 |
| Lateral Corticospinal Tract | Right | C5 | 0.00040 ± 0.00035 | 0.00042 ± 0.00011 | 0.849246 | 1 | 0.051 |
| Abbreviations: FA = fractional anisotropy; MD = mean diffusivity; AD = axial diffusivity; RD = radial diffusivity; SMA = spinal muscular atrophy. Group 1 = SMA patients; Group 2 = healthy controls.. | | | | | | | |

| Supplementary Table 2. Longitudinal changes in spinal cord MRI parameters between baseline and follow-up ( 12–14 months) in adult patients with 5q-SMA. Grey matter cross-sectional area (GM CSA) and DTI metrics (FA, MD, AD, RD) are shown for all cervical levels (C2–C5) and white matter tracts. | | | | | | | |
| --- | --- | --- | --- | --- | --- | --- | --- |
| Metric / Structure | Side | Level | Baseline (mean ± SD) | Follow up (mean ± SD) | p (uncorrected) | p (Bonferroni) | Cohen's d |
| Grey Matter Cross-Sectional Area (GM CSA) | | | | | | | |
| GM CSA (mm²) | — | — | 11.90162 ± 2.55526 mm² | 12.16483 ± 2.70891 mm² | 0.731 | 0.731 | 0.10 |
| FA | | | | | | | |
| Fasciculus Gracilis | Left | C2 | 0.77079 ± 0.07008 | 0.76797 ± 0.04926 | 0.875 | 1.000 | 0.05 |
| Fasciculus Gracilis | Left | C3 | 0.77717 ± 0.06368 | 0.79837 ± 0.04975 | 0.211 | 1.000 | 0.37 |
| Fasciculus Gracilis | Left | C4 | 0.79358 ± 0.04866 | 0.79594 ± 0.05331 | 0.873 | 1.000 | 0.05 |
| Fasciculus Gracilis | Left | C5 | 0.78317 ± 0.07098 | 0.77522 ± 0.07578 | 0.731 | 1.000 | 0.11 |
| Fasciculus Gracilis | Right | C2 | 0.76327 ± 0.09822 | 0.76684 ± 0.07226 | 0.888 | 1.000 | 0.04 |
| Fasciculus Gracilis | Right | C3 | 0.76365 ± 0.07318 | 0.77901 ± 0.04247 | 0.390 | 1.000 | 0.25 |
| Fasciculus Gracilis | Right | C4 | 0.78086 ± 0.06272 | 0.79167 ± 0.05980 | 0.546 | 1.000 | 0.18 |
| Fasciculus Gracilis | Right | C5 | 0.78044 ± 0.07924 | 0.76401 ± 0.08381 | 0.523 | 1.000 | 0.20 |
| Fasciculus Cuneatus | Left | C2 | 0.83508 ± 0.10187 | 0.83631 ± 0.04941 | 0.959 | 1.000 | 0.01 |
| Fasciculus Cuneatus | Left | C3 | 0.83906 ± 0.07274 | 0.85874 ± 0.04476 | 0.276 | 1.000 | 0.32 |
| Fasciculus Cuneatus | Left | C4 | 0.82367 ± 0.05671 | 0.81577 ± 0.05098 | 0.617 | 1.000 | 0.15 |
| Fasciculus Cuneatus | Left | C5 | 0.78871 ± 0.08389 | 0.78555 ± 0.06503 | 0.895 | 1.000 | 0.04 |
| Fasciculus Cuneatus | Right | C2 | 0.82924 ± 0.08244 | 0.83938 ± 0.04840 | 0.614 | 1.000 | 0.15 |
| Fasciculus Cuneatus | Right | C3 | 0.83197 ± 0.08129 | 0.85468 ± 0.03883 | 0.237 | 1.000 | 0.35 |
| Fasciculus Cuneatus | Right | C4 | 0.83417 ± 0.06635 | 0.82449 ± 0.07497 | 0.637 | 1.000 | 0.14 |
| Fasciculus Cuneatus | Right | C5 | 0.78399 ± 0.06999 | 0.77032 ± 0.07339 | 0.546 | 1.000 | 0.19 |
| Lateral Corticospinal Tract | Left | C2 | 0.75454 ± 0.06421 | 0.75450 ± 0.06243 | 0.999 | 1.000 | 0.00 |
| Lateral Corticospinal Tract | Left | C3 | 0.76498 ± 0.06231 | 0.76681 ± 0.04626 | 0.910 | 1.000 | 0.03 |
| Lateral Corticospinal Tract | Left | C4 | 0.74086 ± 0.06136 | 0.73554 ± 0.04889 | 0.745 | 1.000 | 0.09 |
| Lateral Corticospinal Tract | Left | C5 | 0.73600 ± 0.07100 | 0.70207 ± 0.10529 | 0.228 | 1.000 | 0.38 |
| Lateral Corticospinal Tract | Right | C2 | 0.75256 ± 0.07783 | 0.76308 ± 0.05552 | 0.599 | 1.000 | 0.15 |
| Lateral Corticospinal Tract | Right | C3 | 0.74638 ± 0.07929 | 0.71578 ± 0.06710 | 0.174 | 1.000 | 0.42 |
| Lateral Corticospinal Tract | Right | C4 | 0.74190 ± 0.04368 | 0.77406 ± 0.04572 | 0.013 | 0.319 | 0.72 |
| Lateral Corticospinal Tract | Right | C5 | 0.71461 ± 0.06855 | 0.73492 ± 0.04730 | 0.245 | 1.000 | 0.35 |
| MD | | | | | | | |
| Fasciculus Gracilis | Left | C2 | 0.00088 ± 0.00015 | 0.00085 ± 0.00010 | 0.572 | 1.000 | 0.17 |
| Fasciculus Gracilis | Left | C3 | 0.00086 ± 0.00008 | 0.00083 ± 0.00009 | 0.304 | 1.000 | 0.30 |
| Fasciculus Gracilis | Left | C4 | 0.00082 ± 0.00008 | 0.00078 ± 0.00008 | 0.120 | 1.000 | 0.46 |
| Fasciculus Gracilis | Left | C5 | 0.00083 ± 0.00011 | 0.00079 ± 0.00014 | 0.312 | 1.000 | 0.32 |
| Fasciculus Gracilis | Right | C2 | 0.00088 ± 0.00016 | 0.00088 ± 0.00012 | 0.974 | 1.000 | 0.01 |
| Fasciculus Gracilis | Right | C3 | 0.00087 ± 0.00009 | 0.00085 ± 0.00008 | 0.598 | 1.000 | 0.16 |
| Fasciculus Gracilis | Right | C4 | 0.00083 ± 0.00007 | 0.00082 ± 0.00008 | 0.601 | 1.000 | 0.15 |
| Fasciculus Gracilis | Right | C5 | 0.00084 ± 0.00012 | 0.00079 ± 0.00014 | 0.236 | 1.000 | 0.38 |
| Fasciculus Cuneatus | Left | C2 | 0.00080 ± 0.00015 | 0.00078 ± 0.00008 | 0.654 | 1.000 | 0.13 |
| Fasciculus Cuneatus | Left | C3 | 0.00075 ± 0.00009 | 0.00075 ± 0.00009 | 0.923 | 1.000 | 0.03 |
| Fasciculus Cuneatus | Left | C4 | 0.00074 ± 0.00007 | 0.00073 ± 0.00008 | 0.412 | 1.000 | 0.24 |
| Fasciculus Cuneatus | Left | C5 | 0.00074 ± 0.00015 | 0.00069 ± 0.00012 | 0.193 | 1.000 | 0.41 |
| Fasciculus Cuneatus | Right | C2 | 0.00083 ± 0.00016 | 0.00080 ± 0.00007 | 0.448 | 1.000 | 0.22 |
| Fasciculus Cuneatus | Right | C3 | 0.00079 ± 0.00012 | 0.00075 ± 0.00009 | 0.325 | 1.000 | 0.29 |
| Fasciculus Cuneatus | Right | C4 | 0.00075 ± 0.00007 | 0.00077 ± 0.00014 | 0.620 | 1.000 | 0.15 |
| Fasciculus Cuneatus | Right | C5 | 0.00076 ± 0.00012 | 0.00070 ± 0.00009 | 0.107 | 1.000 | 0.52 |
| Lateral Corticospinal Tract | Left | C2 | 0.00085 ± 0.00014 | 0.00084 ± 0.00009 | 0.808 | 1.000 | 0.07 |
| Lateral Corticospinal Tract | Left | C3 | 0.00082 ± 0.00012 | 0.00082 ± 0.00008 | 0.963 | 1.000 | 0.01 |
| Lateral Corticospinal Tract | Left | C4 | 0.00083 ± 0.00007 | 0.00081 ± 0.00010 | 0.433 | 1.000 | 0.23 |
| Lateral Corticospinal Tract | Left | C5 | 0.00084 ± 0.00010 | 0.00082 ± 0.00013 | 0.656 | 1.000 | 0.14 |
| Lateral Corticospinal Tract | Right | C2 | 0.00087 ± 0.00017 | 0.00084 ± 0.00007 | 0.548 | 1.000 | 0.18 |
| Lateral Corticospinal Tract | Right | C3 | 0.00084 ± 0.00013 | 0.00083 ± 0.00007 | 0.681 | 1.000 | 0.12 |
| Lateral Corticospinal Tract | Right | C4 | 0.00084 ± 0.00007 | 0.00082 ± 0.00007 | 0.309 | 1.000 | 0.30 |
| Lateral Corticospinal Tract | Right | C5 | 0.00085 ± 0.00010 | 0.00079 ± 0.00010 | 0.091 | 1.000 | 0.54 |
| AD | | | | | | | |
| Fasciculus Gracilis | Left | C2 | 0.00196 ± 0.00027 | 0.00190 ± 0.00011 | 0.281 | 1.000 | 0.32 |
| Fasciculus Gracilis | Left | C3 | 0.00191 ± 0.00015 | 0.00189 ± 0.00012 | 0.666 | 1.000 | 0.13 |
| Fasciculus Gracilis | Left | C4 | 0.00186 ± 0.00016 | 0.00179 ± 0.00020 | 0.156 | 1.000 | 0.42 |
| Fasciculus Gracilis | Left | C5 | 0.00183 ± 0.00015 | 0.00173 ± 0.00024 | 0.119 | 1.000 | 0.50 |
| Fasciculus Gracilis | Right | C2 | 0.00195 ± 0.00022 | 0.00195 ± 0.00016 | 0.952 | 1.000 | 0.02 |
| Fasciculus Gracilis | Right | C3 | 0.00189 ± 0.00016 | 0.00189 ± 0.00013 | 0.915 | 1.000 | 0.03 |
| Fasciculus Gracilis | Right | C4 | 0.00186 ± 0.00015 | 0.00185 ± 0.00013 | 0.722 | 1.000 | 0.10 |
| Fasciculus Gracilis | Right | C5 | 0.00185 ± 0.00019 | 0.00169 ± 0.00024 | 0.024 | 0.585 | 0.73 |
| Fasciculus Cuneatus | Left | C2 | 0.00197 ± 0.00022 | 0.00189 ± 0.00013 | 0.177 | 1.000 | 0.40 |
| Fasciculus Cuneatus | Left | C3 | 0.00184 ± 0.00022 | 0.00185 ± 0.00013 | 0.727 | 1.000 | 0.10 |
| Fasciculus Cuneatus | Left | C4 | 0.00177 ± 0.00015 | 0.00170 ± 0.00018 | 0.125 | 1.000 | 0.46 |
| Fasciculus Cuneatus | Left | C5 | 0.00167 ± 0.00022 | 0.00154 ± 0.00022 | 0.064 | 1.000 | 0.60 |
| Fasciculus Cuneatus | Right | C2 | 0.00199 ± 0.00023 | 0.00193 ± 0.00012 | 0.286 | 1.000 | 0.32 |
| Fasciculus Cuneatus | Right | C3 | 0.00189 ± 0.00021 | 0.00185 ± 0.00012 | 0.507 | 1.000 | 0.20 |
| Fasciculus Cuneatus | Right | C4 | 0.00181 ± 0.00013 | 0.00179 ± 0.00013 | 0.576 | 1.000 | 0.16 |
| Fasciculus Cuneatus | Right | C5 | 0.00169 ± 0.00019 | 0.00155 ± 0.00015 | 0.011 | 0.273 | 0.83 |
| Lateral Corticospinal Tract | Left | C2 | 0.00181 ± 0.00020 | 0.00179 ± 0.00013 | 0.810 | 1.000 | 0.07 |
| Lateral Corticospinal Tract | Left | C3 | 0.00176 ± 0.00021 | 0.00175 ± 0.00012 | 0.833 | 1.000 | 0.06 |
| Lateral Corticospinal Tract | Left | C4 | 0.00173 ± 0.00012 | 0.00166 ± 0.00022 | 0.168 | 1.000 | 0.41 |
| Lateral Corticospinal Tract | Left | C5 | 0.00170 ± 0.00012 | 0.00159 ± 0.00020 | 0.046 | 1.000 | 0.64 |
| Lateral Corticospinal Tract | Right | C2 | 0.00182 ± 0.00023 | 0.00181 ± 0.00011 | 0.766 | 1.000 | 0.09 |
| Lateral Corticospinal Tract | Right | C3 | 0.00176 ± 0.00024 | 0.00176 ± 0.00011 | 0.919 | 1.000 | 0.03 |
| Lateral Corticospinal Tract | Right | C4 | 0.00173 ± 0.00012 | 0.00168 ± 0.00010 | 0.132 | 1.000 | 0.45 |
| Lateral Corticospinal Tract | Right | C5 | 0.00169 ± 0.00010 | 0.00157 ± 0.00016 | 0.010 | 0.230 | 0.85 |
| RD | | | | | | | |
| Fasciculus Gracilis | Left | C2 | 0.00035 ± 0.00018 | 0.00033 ± 0.00011 | 0.714 | 1.000 | 0.11 |
| Fasciculus Gracilis | Left | C3 | 0.00035 ± 0.00014 | 0.00030 ± 0.00010 | 0.157 | 1.000 | 0.42 |
| Fasciculus Gracilis | Left | C4 | 0.00031 ± 0.00011 | 0.00028 ± 0.00008 | 0.283 | 1.000 | 0.31 |
| Fasciculus Gracilis | Left | C5 | 0.00033 ± 0.00013 | 0.00032 ± 0.00014 | 0.937 | 1.000 | 0.02 |
| Fasciculus Gracilis | Right | C2 | 0.00037 ± 0.00022 | 0.00035 ± 0.00014 | 0.739 | 1.000 | 0.10 |
| Fasciculus Gracilis | Right | C3 | 0.00038 ± 0.00020 | 0.00033 ± 0.00009 | 0.309 | 1.000 | 0.30 |
| Fasciculus Gracilis | Right | C4 | 0.00032 ± 0.00011 | 0.00030 ± 0.00010 | 0.483 | 1.000 | 0.20 |
| Fasciculus Gracilis | Right | C5 | 0.00032 ± 0.00012 | 0.00033 ± 0.00016 | 0.726 | 1.000 | 0.11 |
| Fasciculus Cuneatus | Left | C2 | 0.00026 ± 0.00028 | 0.00023 ± 0.00010 | 0.629 | 1.000 | 0.14 |
| Fasciculus Cuneatus | Left | C3 | 0.00022 ± 0.00014 | 0.00019 ± 0.00010 | 0.382 | 1.000 | 0.26 |
| Fasciculus Cuneatus | Left | C4 | 0.00025 ± 0.00015 | 0.00024 ± 0.00008 | 0.666 | 1.000 | 0.13 |
| Fasciculus Cuneatus | Left | C5 | 0.00027 ± 0.00015 | 0.00026 ± 0.00011 | 0.899 | 1.000 | 0.04 |
| Fasciculus Cuneatus | Right | C2 | 0.00028 ± 0.00024 | 0.00023 ± 0.00009 | 0.398 | 1.000 | 0.25 |
| Fasciculus Cuneatus | Right | C3 | 0.00027 ± 0.00023 | 0.00020 ± 0.00009 | 0.226 | 1.000 | 0.36 |
| Fasciculus Cuneatus | Right | C4 | 0.00024 ± 0.00016 | 0.00025 ± 0.00017 | 0.762 | 1.000 | 0.09 |
| Fasciculus Cuneatus | Right | C5 | 0.00027 ± 0.00012 | 0.00028 ± 0.00011 | 0.911 | 1.000 | 0.04 |
| Lateral Corticospinal Tract | Left | C2 | 0.00039 ± 0.00015 | 0.00037 ± 0.00011 | 0.663 | 1.000 | 0.13 |
| Lateral Corticospinal Tract | Left | C3 | 0.00038 ± 0.00015 | 0.00036 ± 0.00008 | 0.613 | 1.000 | 0.15 |
| Lateral Corticospinal Tract | Left | C4 | 0.00042 ± 0.00018 | 0.00039 ± 0.00008 | 0.475 | 1.000 | 0.21 |
| Lateral Corticospinal Tract | Left | C5 | 0.00039 ± 0.00013 | 0.00043 ± 0.00016 | 0.400 | 1.000 | 0.27 |
| Lateral Corticospinal Tract | Right | C2 | 0.00039 ± 0.00018 | 0.00036 ± 0.00009 | 0.451 | 1.000 | 0.22 |
| Lateral Corticospinal Tract | Right | C3 | 0.00042 ± 0.00022 | 0.00037 ± 0.00007 | 0.322 | 1.000 | 0.29 |
| Lateral Corticospinal Tract | Right | C4 | 0.00040 ± 0.00009 | 0.00038 ± 0.00010 | 0.597 | 1.000 | 0.15 |
| Lateral Corticospinal Tract | Right | C5 | 0.00042 ± 0.00011 | 0.00040 ± 0.00013 | 0.762 | 1.000 | 0.10 |
| Abbreviations: FA = fractional anisotropy; MD = mean diffusivity; AD = axial diffusivity; RD = radial diffusivity; GM CSA = grey matter cross-sectional area; SMA = spinal muscular atrophy. | | | | | | | |
